# Supplementary material for: JOINTLY: interpretable joint clustering of single-cell transcriptomes
Source: Nat Commun. 2023 Dec 20;14:8473. doi: 10.1038/s41467-023-44279-8 (PMC10733431; doi:10.1038/s41467-023-44279-8)
Supplement: Supplementary file 1 — Supplementary Information [file 41467_2023_44279_MOESM1_ESM.pdf]

JONTLY: Interpretable joint clustering of single-cell transcriptomes

Andreas Fønss Møller[1,2], Jesper Grud Skat Madsen[1,3,4,5]*

Affiliations:

[1]Institute of Biochemistry and Molecular Biology, University of Southern, Denmark

[2]Sino-Danish College (SDC), University of Chinese Academy of Sciences, China

[3]Institute of Mathematics and Computer Science, University of Southern Denmark

[4]Center for Functional Genomics and Tissue Plasticity (ATLAS), Odense M, 5230, Denmark

[5]The Novo Nordisk Foundation Center for Genomic Mechanisms of Disease, Broad Institute of MIT and Harvard, Cambridge, MA 02142, USA

*Corresponding author. Email: jgsm@imada.sdu.dk

Supplementary Note 1: Consensus PCA

As a preprocessing step for the JOINTLY model, we implement a flexible variant of consensus PCA, that captures common variation and flexibility incorporates dataset-specific variation. For normalised and standardised gene expression values $X_d = (x_1, x_2, \dots x_n) \in \mathbb{R}^{mxn}$ for *m* highly variable genes and *n* cells in dataset *d*, we calculate the variance-covariance matrix:

$$C_d = \frac{(X_d * X_d^T)}{n_d - 1} \tag{1}$$

For each dataset, *d*, calculate the variance-covariance ($C_d \in \mathbb{R}^{mxm}$) by matrix multiplication of the normalised and standardised gene expression values (X_d) and adjusting for the number cells (n_d) – 1. This matrix captures the variance of each feature along the diagonal and the covariance between each pair of features off the diagonal. Next, we calculate the within-group variance-covariance matrix ($C_g \in \mathbb{R}^{mxm}$) by summing the variance-covariance matrices for each dataset (C_d) multiplied by the number of cells in each dataset (n_d) and divided by the total number of cells across all datasets:

$$C_g = \sum_{d=1}^{D} \frac{C_d * n_d}{\sum_{d=1}^{D} n_d} \tag{2}$$

The within-group variance-covariance matrix is the decomposed into a reduced dimensional space of *k* dimensions using randomised singular value decomposition[1]. By matrix multiplication between the left singular vectors ($U \in \mathbb{R}^{mxk}$) and the transposed normalised and standardised gene expression values for each dataset, we calculate a reduced dimensional space for cells.

$$E_d = U^T * X_d \tag{3}$$

The embedding matrices ($E_d \in \mathbb{R}^{kxn}$) are then used to calculate the fraction of variance explained in the common reduced dimensional space relative to a dataset-specific dimensional reduction. For datasets,

where the common reduced space explains less than 80% of the variance explained by the dataset-specific reduced space, we calculate additional singular vectors. First, we calculate the residual matrix between the variance-covariance matrix (C_g) and the reduced dimensional space using the left singular vectors ($U \in \mathbb{R}^{mxk}$)

$$R_g = C_g - U * U^T * C_g \tag{4}$$

The residual matrices ($R_g \in \mathbb{R}^{mxm}$) are subject to randomised SVD as described above and dataset-specific left singular vectors are concatenated to the U matrix, such that U describes at least 80% variance in each dataset, resulting in the matrix $U_{\text{all}} = (U, U_d)$ which is a m x k_{all} sized matrix, where k_{all} is equal to the initially chosen k and any additional dataset-specific vectors. Finally, the consensus PCA space is calculated by matrix multiplication between U and the normalised and standardised gene expression values:

$$E_d = U_{\text{all}}^T * X_d \tag{5}$$

These embedding matrices ($E_d \in \mathbb{R}^{k_{\text{all}}xn}$) are used to generate kernel matrices ($K_d \in \mathbb{R}^{nxn}$) using an alpha-decay kernel[2] as well as adjacency ($A_d \in \mathbb{R}^{nxn}$) and degree ($L_d \in \mathbb{R}^{nxn}$) matrices, which are calculated from a shared nearest neighbours graph built on the embedding matrices.

Supplementary Note 2: The JOINTLY algorithm

Graph regularised kernel non-negative matrix factorisation in general terms

We have implemented graph regularised kernel non-negative matrix factorisation (NMF) from KOGNMF[3]. Briefly, let $X = (x_1, x_2, \dots x_n) \in \mathbb{R}^{mxn}$ as a data matrix of non-negative elements. Regular NMF factorises X into two low-rank non-negative matrices:

$$X \approx VH \tag{6}$$

where H is the clustering matrix defined as $H^T = (h_1, h_2, \dots h_k) \in \mathbb{R}^{kxn}$, V is the basis matrix defined as $V = (v_1, v_2, \dots v_k) \in \mathbb{R}^{mxk}$, and k is the factorisation rank, which is generally much smaller than the smallest dimension of X. This problem can be solved using gradient descent to minimise the reconstruction error under non-negative constraints[4].

To obtain a non-linear factorisation, a non-linear transformation of X to a higher or infinite O dimensional space, such that $\Phi(X) = (\Phi(x_1), \Phi(x_2), \dots \Phi(x_n)) \in \mathbb{R}^{Oxn}$ is introduced. This can also factorise this non-linear space into two low-rank matrices:

$$\Phi(X) \approx WH \tag{7}$$

To solve this problem, the following loss function can be defined:

$$\underset{W,H}{\text{argmin}} = \left|\left|\Phi(X) - WH\right|\right| \tag{8}$$

However, this problem cannot directly be minimised since $\Phi(X)$ potentially has infinite dimensions[3]. This issue can be circumvented by using the kernel trick by defining that W is a linear combination of the features in the non-linear transformed space $\Phi(X)$. The linear combinations are defined by a new matrix called F, such that:

$$W = \Phi(X)F \tag{9}$$

Substitution of this into the loss function obtains:

$$\underset{F,H}{\text{argmin}} = \left|\left|\Phi(X) - \Phi(X)FH\right|\right| \tag{10}$$

Now, let $K \in \mathbb{R}^{nxn}$ be a kernel matrix such that $K = \Phi^T(X)\Phi(X)$. This can be exploited to derive multiplicative updating rules for updating H and F without factorising $\Phi(X)$ with weights α and μ:

$$H \leftarrow H \odot \frac{\alpha F^T K + 2\mu H}{\alpha F^T K F H + 2\mu H H^T H} \tag{11}$$

$$F \leftarrow F \odot \frac{KH^T}{KFHH^T} \tag{12}$$

To add graph regularisation, such that the factorisation also reconstructs the geometric structure of the data in the non-linear feature space, the following loss function is introduced:

$$\underset{F,H}{\operatorname{argmin}} = \alpha\left|\left|\Phi(X) - \Phi(X)FH\right|\right| + \lambda\mathrm{Tr}(HLH^T) \tag{13}$$

where L is the Laplacian matrix defined as $L = D - A$ where D is the degree matrix and A is the adjacency matrix of the graph resulting in the following update rules:

$$H \leftarrow H \odot \frac{\alpha F^T K + 2\mu H + \lambda HA}{\alpha F^T KFH + 2\mu HH^T H + \lambda HD} \tag{14}$$

$$F \leftarrow F \odot \frac{KH^T}{KFHH^T} \tag{15}$$

Extending KOGNMF for joint clustering of single-cell RNA-sequence datasets

In the case of single-cell RNA-sequence datasets, this loss function optimises two low-rank matrices per dataset; the clustering matrix ($H_d \in \mathbb{R}^{fxn}$) and the basis matrix in kernel space ($D_d \in \mathbb{R}^{nxF}$) where f is the number of factors and n is the number of cells. The required inputs are a kernel matrix ($K_d \in \mathbb{R}^{nxn}$), an adjacency ($A_d \in \mathbb{R}^{nxn}$), and a degree matrix ($L_d \in \mathbb{R}^{nxn}$), all of which are calculated using consensus PCA space (see Methods and Supplementary Note 1).

The graph-regularised kernel NMF can factorise a single dataset considering non-linear similarities between data points. In the case of joint clustering of single-cell RNA-sequencing datasets, we have multiple datasets, and there are no guarantees that the same features would contribute to the same factors if the datasets were factorised independently. To solve this and learn a clustering matrix, H, where the factors are explained by similar factors across datasets, JOINTLY introduces a feature matrix $V_d = (v_1, v_2, \dots v_k) \in \mathbb{R}^{mxk}$ per dataset, d where m is the number of highly variable genes. This matrix is factorised with the clustering matrix, H_d, resembling regular NMF and reconstructing the original data matrix as $X_d \approx V_d H_d$. To use this for minimising the difference between factors across datasets, JOINTLY introduces a loss based on the difference between the reconstructed gene expression space using the V matrix for the target dataset and all other datasets, minimising the difference in reconstruction between V matrices:

$$\underset{F,H}{\operatorname{argmin}} = \alpha\left|\left|\Phi(X_d) - \Phi(X_d)F_d H_d\right|\right| + \lambda\mathrm{Tr}\left(H_d L_d H_d^T\right) + \beta \sum_{d=1}^{D}\sum_{j \neq d}\left|\left|V_j H_d - V_d H_d\right|\right| \tag{16}$$

For each dataset, this gives us the following update rules for H and F with weights α, μ, λ and β:

$$H_d \leftarrow H_d \odot \frac{\alpha F_d^T K_d + 2\mu H_d + \lambda H_d A_d + \sum_{j \neq d} \beta V_j^T X_d + \beta V_d^T V_d H_d}{\alpha F_d^T K_d F_d H_d + 2\mu H_d H_d^T H_d + \lambda H_d D_d + \sum_{j \neq d} \beta V_j^T V_j H_d + 2\beta V_d^T V_j H_d + \beta V_d^T X_d} \quad (17)$$

$$F_d \leftarrow F_d \odot \frac{K_d H_d^T}{K_d F_d H_d H_d^T} \quad (18)$$

After updating H and F for all datasets, we update V for each dataset using the new H and least squares setting negative values to 0:

$$V_d = (X_d X_d^T)^{-1} X_d^T H_d \quad (19)$$

Table 1: Summary of symbols

Symbol	Description	Shape
d, j	Dataset identifier	
n	Number of genes	scalar
m	Number of cells	scalar
k	Rank for a lower-dimensional space	scalar
X	Normalised and standardised gene expression values	m x n
C	Variance-covariance matrix	m x m
C_g	Within-group variance-covariance matrix	m x m
U	Left singular vectors	m x k
R	Residuals	m x m
H	Clustering matrix	k x n
W	Coefficient matrix in non-linear space	o x k
V	Coefficient matrix	m x k
F	Coefficient matrix	n x k
K	Kernel matrix	n x n
L	Graph Laplacian	n x n
A	Adjacency matrix	n x n
D	Degree matrix	n x n
Φ	Non-linear mapping function	function
α	JOINTLY loss weight	scalar
β	JOINTLY loss weight	scalar
μ	JOINTLY loss weight	scalar
λ	JOINTLY loss weight	scalar
O	Higher-dimensional or infinitely dimensional space	scalar

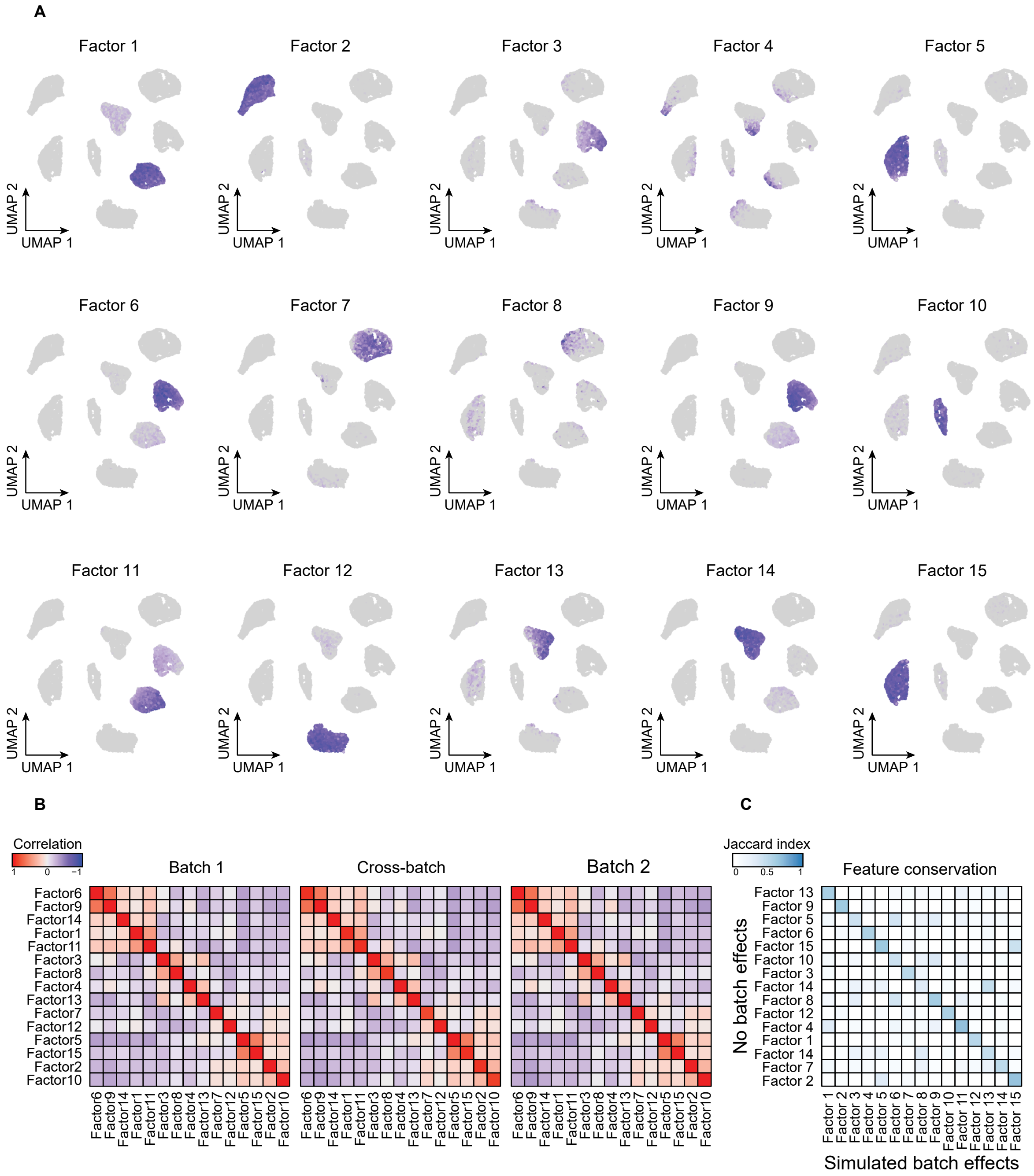

Supplementary Figure 1: JOINTLY factors generalize across batches.
A) UMAPs coloured by module scores derived from JOINTLY factors in cell lines with simulated batch effects. **B**) Heatmap showing pairwise correlation of modules scores derived JOINTLY factor, within batches and between batches. **C**) Heatmap showing pairwise Jaccard index between factor gene sets from the cell lines with and without simulated batch effects. Source data are provided as a Source Data file.

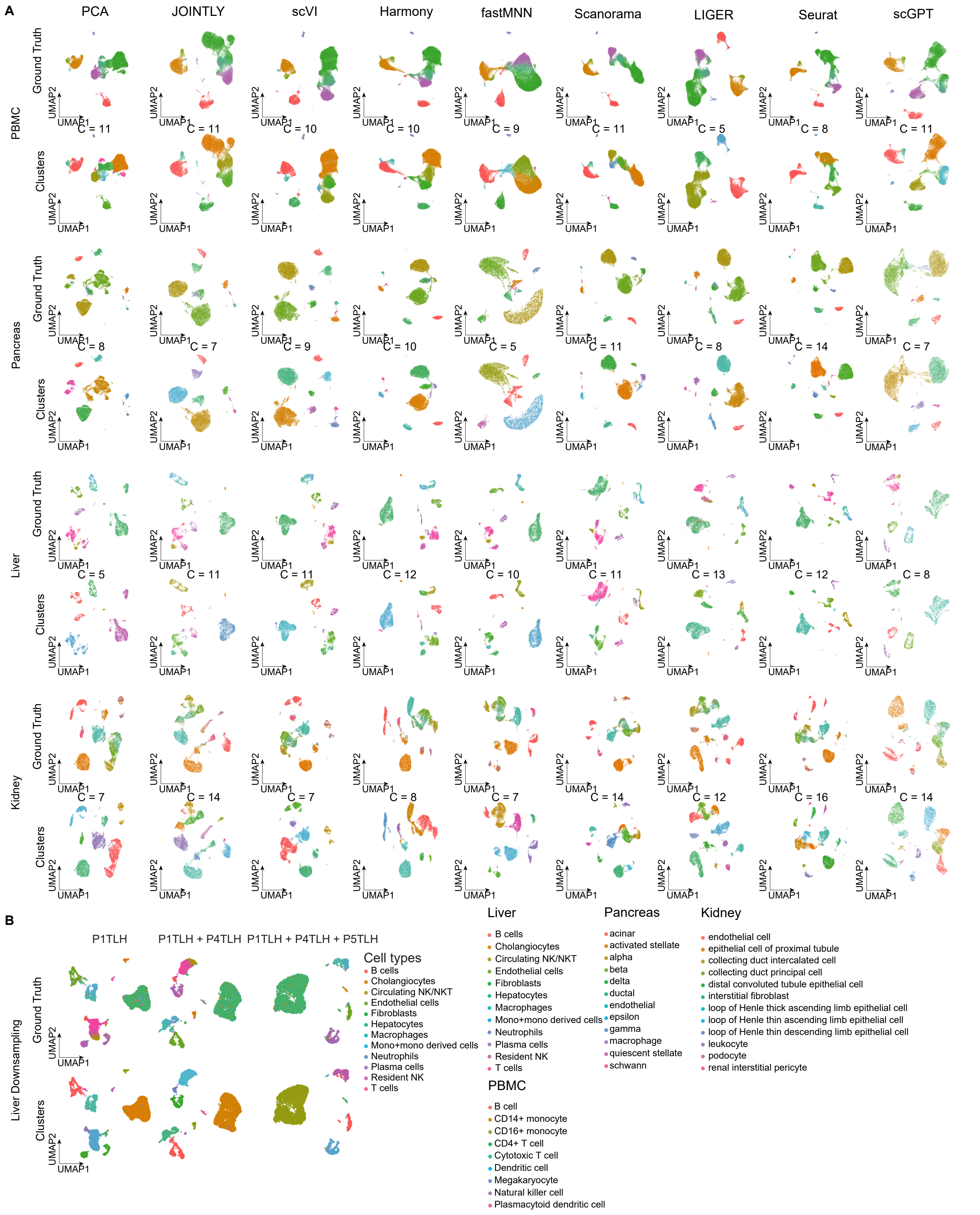

Supplementary Figure 2: Embeddings of integrated datasets.
A) UMAP based on the embedded space for the indicated method and dataset, coloured by transferred cell type labels (see Methods) and cluster labels. The number of clusters is noted on the plot. **B**) UMAPs of JOINTLY embeddings of human liver atlas with the indicated samples removed.

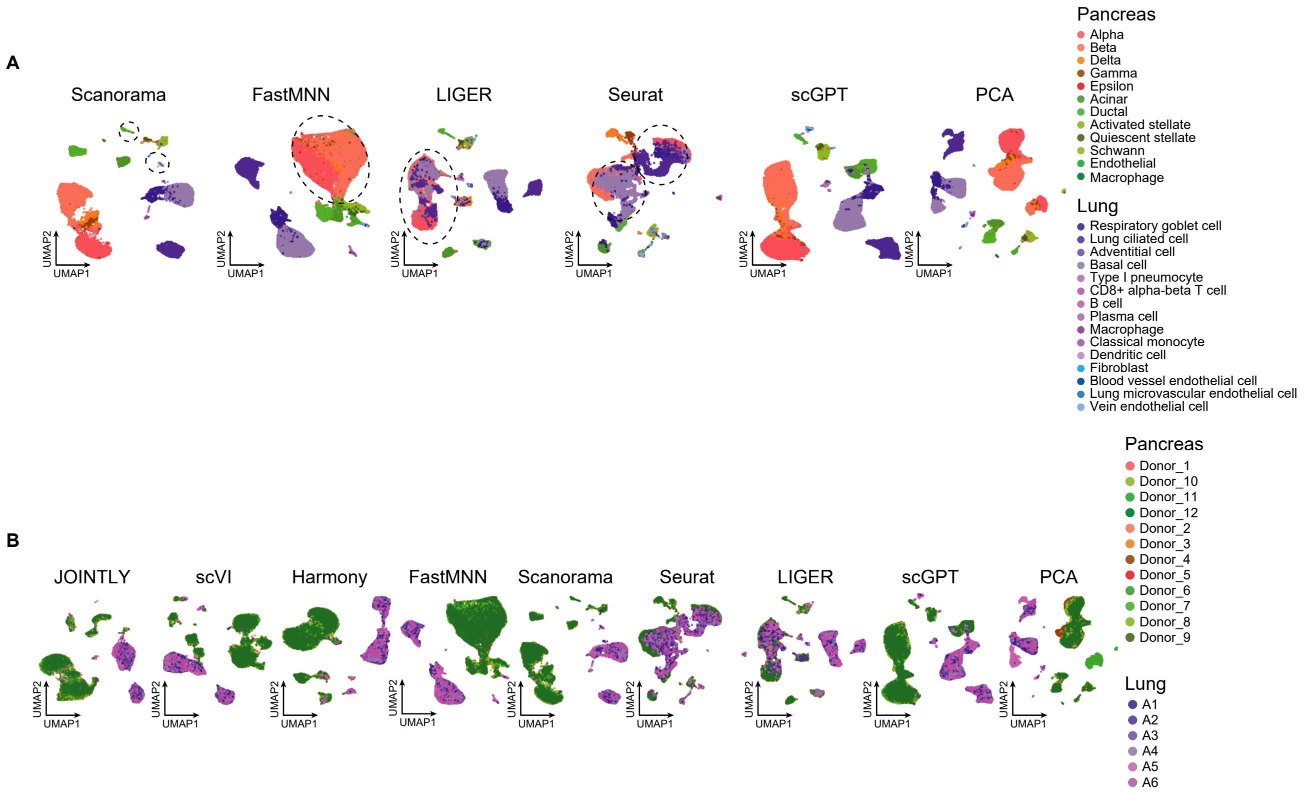

Supplementary Figure 3: Integration of biologically diverse datasets.
A) UMAPs coloured by transferred cell type labels (see Methods) based on the embedded space for the indicated method for the combined Pancreas and Lung dataset. **B**) UMAPs coloured by batch labels based on the embedded space for the indicated method for the combined Pancreas and Lung dataset.

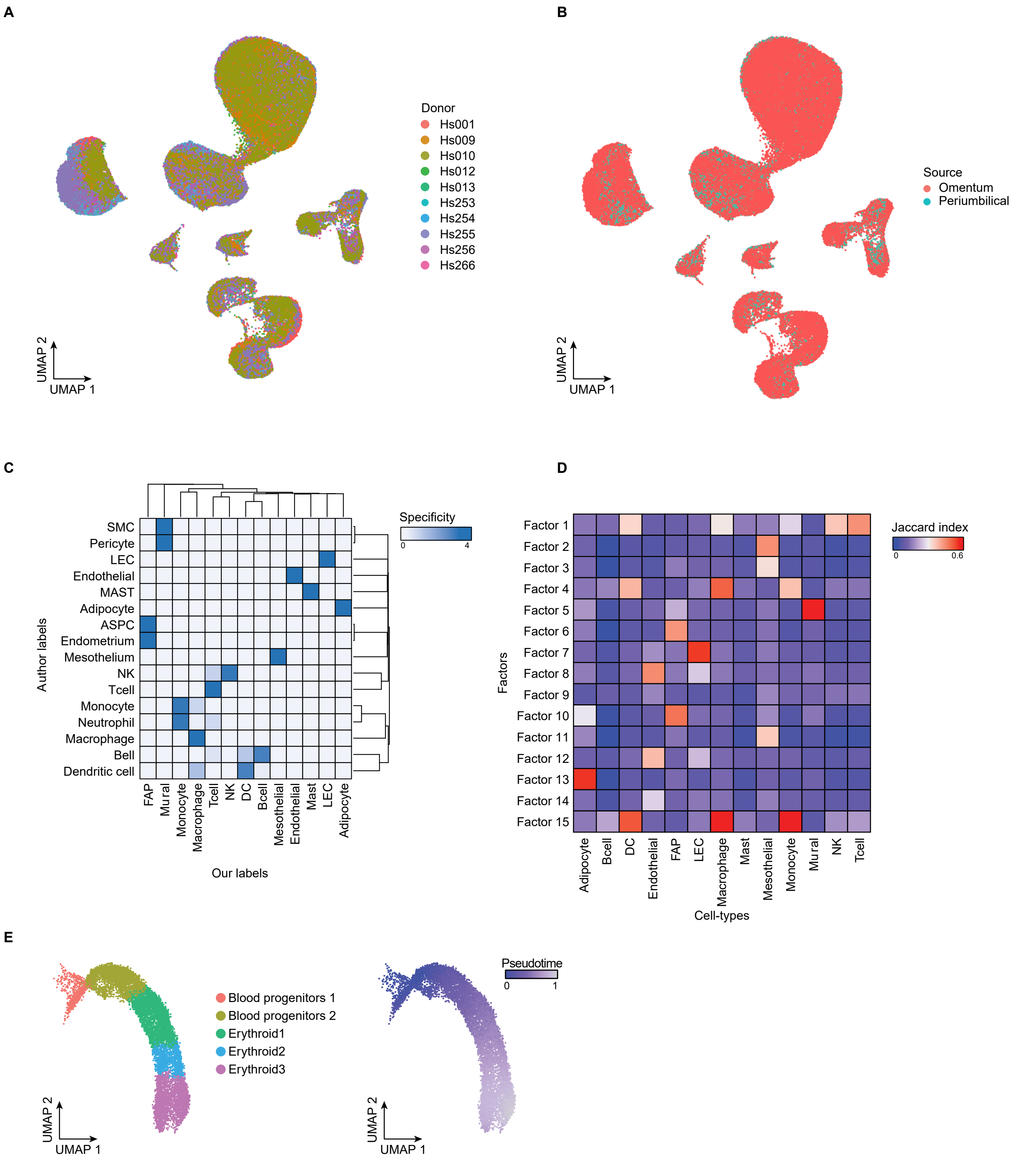

Supplementary Figure 4: JOINTLY integration of adipose tissue.
A-B) UMAP based on JOINTLY embedding of adipose tissue atlas from 9 batches[5] coloured by batch (**A**) and tissue source (**B**). **C**) Heatmap showing the specificity between cell type labels identified using JOINTLY and author labels. **D**) Heatmap showing the Jaccard index between marker genes for each cell type and gene assigned to each JOINTLY factor. **E**) UMAP of cells undergoing erythropoiesis during mouse gastrulation[6] coloured by cell type label and scVelo[7] predicted pseudo-time (right). Source data are provided as a Source Data file.

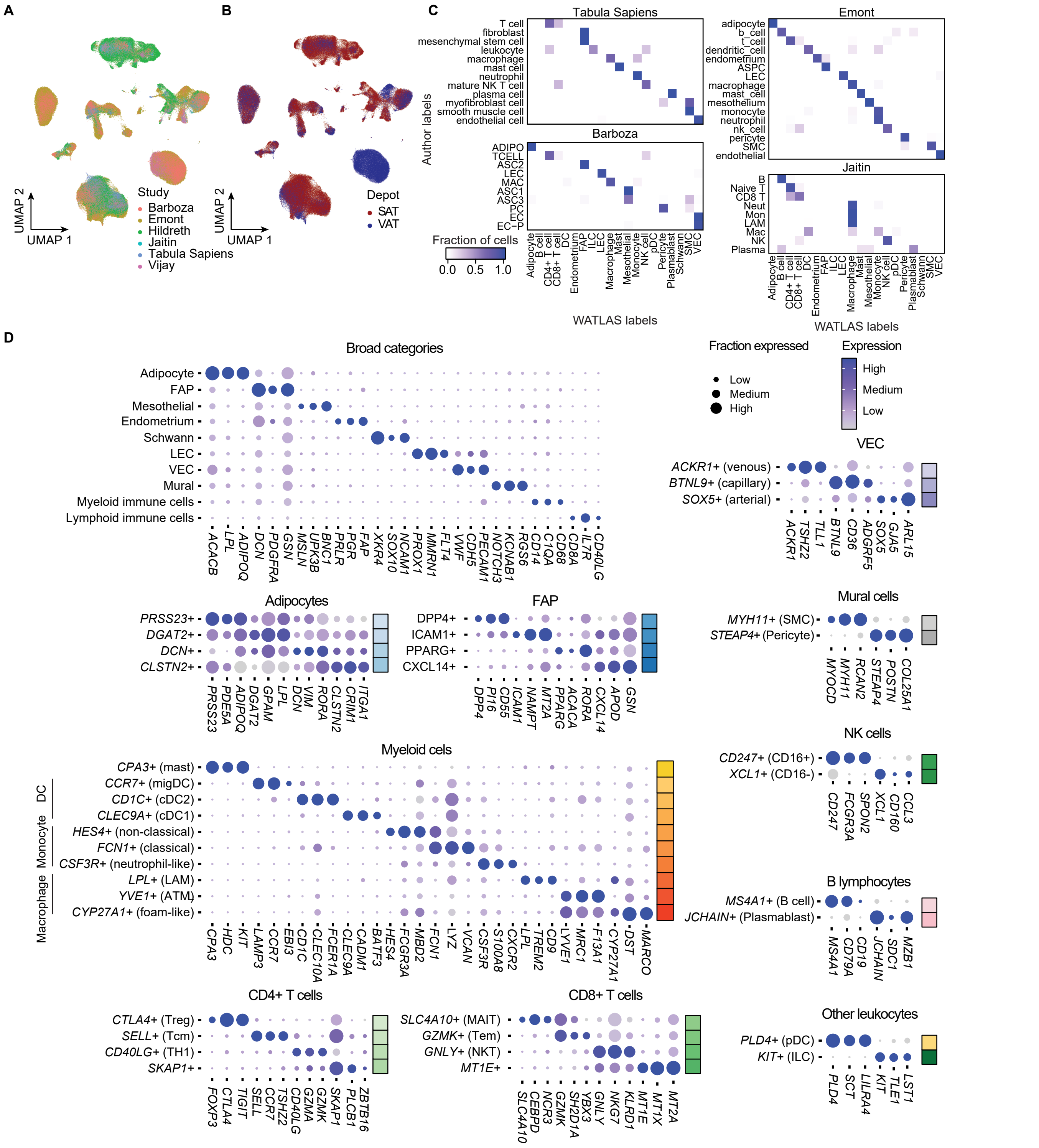

Supplementary Figure 5: WATLAS states marker genes.
A-B) UMAP of WATLAS (see Figure 5A-B) coloured by study (**A**) and adipose depot (**B**). **C**) Heatmap showing the fractional overlap between WATLAS labels and labels defined by the original authors for the indicated studies. **D**) Dotplots showing selected marker genes for the indicated broad cell type categories and sub-types. Source data are provided as a Source Data file.

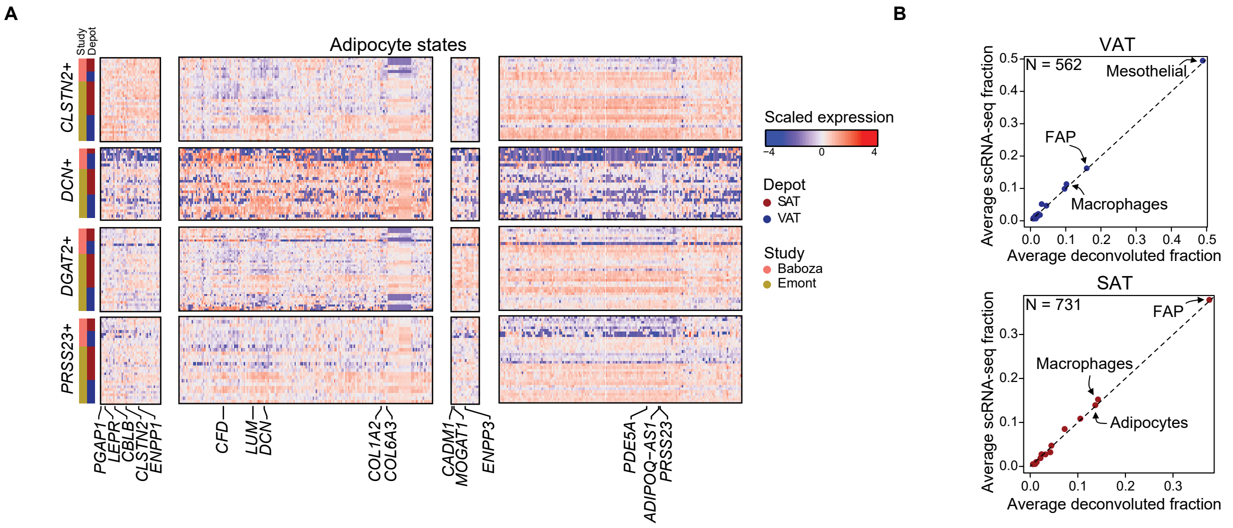

Supplementary Figure 6: WATLAS state modules and decomposition.
A) Heatmap showing normalised and scaled per-donor pseudo-bulk expression levels of genes identified as differentially expressed in the indicated adipocyte populations across all populations, depots, and studies. **B**) Scatterplot showing the relationship between the average fraction of the cell types in WATLAS and the average deconvoluted fractions for VAT and SAT depots (left and right panel respectively). Source data are provided as a Source Data file.

References

1. Halko, N., Martinsson, P.G. & Tropp, J.A. Finding Structure with Randomness: Probabilistic Algorithms for Constructing Approximate Matrix Decompositions. *SIAM Rev.* **53**, 217–288 (2011).
2. Moon, K.R.*, et al.* Visualizing structure and transitions in high-dimensional biological data. *Nature Biotechnology* **37**, 1482-1492 (2019).
3. Tolić, D., Antulov-Fantulin, N. & Kopriva, I. A nonlinear orthogonal non-negative matrix factorization approach to subspace clustering. *Pattern Recognition* **82**, 40-55 (2018).
4. Lee, D. & Seung, H. Algorithms for Non-negative Matrix Factorization. *Adv. Neural Inform. Process. Syst.* **13**(2001).
5. Emont, M.P.*, et al.* A single-cell atlas of human and mouse white adipose tissue. *Nature* **603**, 926-933 (2022).
6. Pijuan-Sala, B.*, et al.* A single-cell molecular map of mouse gastrulation and early organogenesis. *Nature* **566**, 490-495 (2019).
7. Bergen, V., Lange, M., Peidli, S., Wolf, F.A. & Theis, F.J. Generalizing RNA velocity to transient cell states through dynamical modeling. *Nature Biotechnology* **38**, 1408-1414 (2020).
