# Supplementary material for: JOINTLY: interpretable joint clustering of single-cell transcriptomes
Source: Nat Commun. 2023 Dec 20;14:8473. doi: 10.1038/s41467-023-44279-8 (PMC10733431; doi:10.1038/s41467-023-44279-8)
Supplement: Supplementary file 3 — Reporting Summary [file 41467_2023_44279_MOESM3_ESM.pdf]

nature portfolio

Corresponding author(s): Jesper Grud Skat Madsen

Last updated by author(s): Nov 20, 2023

Reporting Summary

Nature Portfolio wishes to improve the reproducibility of the work that we publish. This form provides structure for consistency and transparency in reporting. For further information on Nature Portfolio policies, see our Editorial Policies and the Editorial Policy Checklist.

Statistics

For all statistical analyses, confirm that the following items are present in the figure legend, table legend, main text, or Methods section.

n/a	Confirmed	
☐	☒	The exact sample size (*n*) for each experimental group/condition, given as a discrete number and unit of measurement
☐	☒	A statement on whether measurements were taken from distinct samples or whether the same sample was measured repeatedly
☐	☒	The statistical test(s) used AND whether they are one- or two-sided<br>*Only common tests should be described solely by name; describe more complex techniques in the Methods section.*
☐	☒	A description of all covariates tested
☐	☒	A description of any assumptions or corrections, such as tests of normality and adjustment for multiple comparisons
☐	☒	A full description of the statistical parameters including central tendency (e.g. means) or other basic estimates (e.g. regression coefficient) AND variation (e.g. standard deviation) or associated estimates of uncertainty (e.g. confidence intervals)
☐	☒	For null hypothesis testing, the test statistic (e.g. *F*, *t*, *r*) with confidence intervals, effect sizes, degrees of freedom and *P* value noted<br>*Give P values as exact values whenever suitable.*
☒	☐	For Bayesian analysis, information on the choice of priors and Markov chain Monte Carlo settings
☒	☐	For hierarchical and complex designs, identification of the appropriate level for tests and full reporting of outcomes
☐	☒	Estimates of effect sizes (e.g. Cohen's *d*, Pearson's *r*), indicating how they were calculated

Our web collection on statistics for biologists contains articles on many of the points above.

Software and code

Policy information about availability of computer code

Data collection	R package recount3 version 1.6.0 was used to collect bulk human adipose tissue RNA-seq data from GTEx.
Data analysis	Custom scripts were used to preprocess single cell data.<br>JOINTLY is available as an R package on github at http://www.github.com/madsen-lab/rJOINTLY.<br>Scripts for reproducibility of all analyses are available at: http://www.github.com/madsen-lab/JOINTLY_reproducibility<br>The following open source R packages were used in analysis: Harmony version 1.1.0, rliger version 1.0.1, batchelor version 1.12.3, Seurat version 5.0.0, enrichR version 3.2, HGC version 1.4.0, limma version 3.52.4, gamlss version 5.4-20, Clevr version 0.1.2, Inflection version 1.3.6, emmeans version 1.8.9, bisqueRNA version 1.0.5, compositions version 2.0-6, UCell version 2.0.1, scry version 1.10.0, transformGamPoi 1.2.0, lisi (www.github.com/immunogenomics/LISI) version 1.0, presto (www.github.com/immunogenomics/presto) version 1.0.0, edgeR version 3.40.2.<br>and python packages: scanpy version 1.9.6, scikit-learn version 1.3.2, Scanorama version 1.7.4, scvi-tools version 1.0.4, scgpt version 0.1.6, pandas version 2.1.3, numpy version 1.22.4.

For manuscripts utilizing custom algorithms or software that are central to the research but not yet described in published literature, software must be made available to editors and reviewers. We strongly encourage code deposition in a community repository (e.g. GitHub). See the Nature Portfolio guidelines for submitting code & software for further information.

Data

Policy information about availability of data

All manuscripts must include a data availability statement. This statement should provide the following information, where applicable:

- Accession codes, unique identifiers, or web links for publicly available datasets
- A description of any restrictions on data availability
- For clinical datasets or third party data, please ensure that the statement adheres to our policy

The dataset containing mixtures of cell lines is available through Zenodo under DOI 10.5281/zenodo.3238275 [https://doi.org/10.5281/zenodo.3238275]. The datasets used for benchmarking is available through figshare under DOI 10.6084/m9.figshare.12420968 [https://doi.org/10.6084/m9.figshare.12420968], cellxgene under accession codes bcb61471-2a44-4d00-a0af-ff085512674c [https://cellxgene.cziscience.com/collections/bcb61471-2a44-4d00-a0af-ff085512674c], bd5230f4-cd76-4d35-9ee5-89b3e7475659 [https://cellxgene.cziscience.com/collections/bd5230f4-cd76-4d35-9ee5-89b3e7475659], and 03f821b4-87be-4ff4-b65a-b5fc00061da7 [https://cellxgene.cziscience.com/collections/03f821b4-87be-4ff4-b65a-b5fc00061da7], and NCBI Gene Expression Omnibus under accession code GSE114297 [https://www.ncbi.nlm.nih.gov/geo/query/acc.cgi?acc=GSE114297]. The datasets used for in-vestigating interpretable factors and for building WATLAS are available from the NCBI Gene Expression Omnibus under accession codes GSE128518 [https://www.ncbi.nlm.nih.gov/geo/query/acc.cgi?acc=GSE128518], GSE129363 [https://www.ncbi.nlm.nih.gov/geo/query/acc.cgi?acc=GSE129363], and GSE155960 [https://www.ncbi.nlm.nih.gov/geo/query/acc.cgi?acc=GSE155960] and the Single Cell Portal under accession code SCP1376 [https://singlecell.broadinstitute.org/single_cell/study/SCP1376/a-single-cell-atlas-of-human-and-mouse-white-adipose-tissue]. The processed data for the white adipose tissue atlas is explorable at the Single Cell Portal34 under accession code SCP2289 [https://singlecell.broadinstitute.org/single_cell/study/SCP2289/an-integrated-single-cell-and-single-nucleus-rna-seq-white-adipose-tissue-atlas-watlas]). The model weights for transfer learn-ing and integrating new datasets are available at Zenodo35 under DOI 10.5281/zeodo.8086433 [https://zenodo.org/records/8086433]. The processed datasets used for evaluation and the em-beddings, results, and summaries are available at Zenodo71 under DOI 10.5281/zenodo.8434958 [https://zenodo.org/records/8434958]). Source data are provided with this paper.

Research involving human participants, their data, or biological material

Policy information about studies with human participants or human data. See also policy information about sex, gender (identity/presentation), and sexual orientation and race, ethnicity and racism.

Reporting on sex and gender	In analysis of Emont et al. Adipose data, we report a correlation between a gene factor score and sex.<br>In compositional analysis of our WATLAS, we controlled for sex as a covariate.
Reporting on race, ethnicity, or other socially relevant groupings	Not applicable in this study
Population characteristics	See above
Recruitment	We did not recruit any participants
Ethics oversight	See above

Note that full information on the approval of the study protocol must also be provided in the manuscript.

Field-specific reporting

Please select the one below that is the best fit for your research. If you are not sure, read the appropriate sections before making your selection.

☒ Life sciences ☐ Behavioural & social sciences ☐ Ecological, evolutionary & environmental sciences

For a reference copy of the document with all sections, see nature.com/documents/nr-reporting-summary-flat.pdf

Life sciences study design

All studies must disclose on these points even when the disclosure is negative.

Sample size	No experiments were performed in this study, and therefore the sample sizes were determined by original authors of the public datasets included.
Data exclusions	Benchmarking:<br>All datasets were subset to only contain cells from healthy individuals.<br><br>WATLAS:<br>Cells with an abnormal relationship between the number of detected genes and the total counts were removed in up to two rounds by fitting a linear regression model to the two variables and removing cells with an absolute residual above (Tabula sapiens:2.0, Jaitin: (run22:0.8,run55:0.7), Vijay 1.0, Hildreth: 0.8) in the first round and above (Tabula Sapiens: 0.8) in the second round. Finally, cells with a fraction of counts derived from mitochondrial genes above (Tabula sapiens: 20%, Jaitin: 15%, Vijay: 20%, Hildreth: 15%) where removed. Further, the two diabetic donors from Vijay were removed. After integration, low-quality cells were removed.
Replication	We have demonstrated the use of JOINTLY on real and simulated datasets. All methods in performance benchmark were replicated 5 times

	and the replicate with the best performance were selected for all methods.<br>We have also performed analysis of consistency when down-sampling datasets by 1-3 batches in terms of clustering and integration performance and neighbour consistency.
Randomization	No experiments were performed and therefore no randomization was performed with the exception of applying poisson noise to the CellSIUS dataset. Here cells from each cell line were randomly assigned into two groups, one of which was applied cell line specific batch effects as described in the methods section.
Blinding	No experiments were performed and therefore we were not involved in group allocation. Blinding for group allocation is not possible in the analysis, as group labels was needed for evaluation.

Reporting for specific materials, systems and methods

We require information from authors about some types of materials, experimental systems and methods used in many studies. Here, indicate whether each material, system or method listed is relevant to your study. If you are not sure if a list item applies to your research, read the appropriate section before selecting a response.

Materials & experimental systems

n/a	Involved in the study
☒	☐ Antibodies
☒	☐ Eukaryotic cell lines
☒	☐ Palaeontology and archaeology
☒	☐ Animals and other organisms
☒	☐ Clinical data
☒	☐ Dual use research of concern
☒	☐ Plants

Methods

n/a	Involved in the study
☒	☐ ChIP-seq
☒	☐ Flow cytometry
☒	☐ MRI-based neuroimaging

Plants

Seed stocks	*Report on the source of all seed stocks or other plant material used. If applicable, state the seed stock centre and catalogue number. If plant specimens were collected from the field, describe the collection location, date and sampling procedures.*
Novel plant genotypes	*Describe the methods by which all novel plant genotypes were produced. This includes those generated by transgenic approaches, gene editing, chemical/radiation-based mutagenesis and hybridization. For transgenic lines, describe the transformation method, the number of independent lines analyzed and the generation upon which experiments were performed. For gene-edited lines, describe the editor used, the endogenous sequence targeted for editing, the targeting guide RNA sequence (if applicable) and how the editor was applied.*
Authentication	*Describe any authentication procedures for each seed stock used or novel genotype generated. Describe any experiments used to assess the effect of a mutation and, where applicable, how potential secondary effects (e.g. second site T-DNA insertions, mosiacism, off-target gene editing) were examined.*
